# Metamemory for involuntary autobiographical memories and semantic mind‐pops in 5‐, 7‐ and 9‐year‐old children and young adults

**DOI:** 10.1111/cdev.13794

**Published:** 2022-05-21

**Authors:** Lia Kvavilashvili, Ruth M. Ford

**Affiliations:** ^1^ University of Hertfordshire Hatfield UK; ^2^ Anglia Ruskin University Cambridge UK

## Abstract

In a cross‐sectional study, 5‐, 7‐, and 9‐year‐old‐children and adults (*N* = 144, 86 females, predominantly White U.K. sample of lower‐middle to middle‐class background) were interviewed about their experiences of involuntary autobiographical memories (IAMs) and semantic mind‐pops that come to mind unintentionally. Although some age differences emerged, the majority of participants in all age groups claimed familiarity with involuntary memories and provided examples from their own experience. Moreover, the self‐reported frequency of IAMs and mind‐pops was high, and reported IAMs usually referred to incidental environmental triggers, whereas reported mind‐pops did not. This age invariance highlights the ubiquity of involuntary memories across development and opens up interesting avenues for developmental research on involuntary memories and other spontaneous phenomena (e.g., mind‐wandering, future thinking).

**Abbreviations**
IAM: involuntary autobiographical memories
ToM: theory of mind

Several decades of research on episodic memory has provided ample evidence that when people recall personally experienced events, they engage retrieval processes that are effortful and reconstructive (Schacter & Addis, 2007). Encoding and retrieval of episodic memories involve multiple brain regions, including the medial temporal lobe (especially the hippocampus), posterior parietal cortex, and prefrontal cortex. Neuroimaging evidence suggests that the hippocampus binds the constituent elements of events into coherent encoded representations while the prefrontal cortex supports controlled processes that guide the retrieval of flexibly bound representations (review by Ghetti & Bunge, 2012). Intentional retrieval of information from episodic memory is thus regarded as an act of pattern completion that unifies distributed perceptual and conceptual features (Thompson, 2005). Episodic memory failures and distortions, which are common, are attributed to errors in this complex re‐integrative process (Schacter & Slotnick, 2004).

Unsurprisingly, developmental research has revealed age‐related improvements in voluntary, motivated retrieval of information from episodic memory. As children grow older, they are better able to recall events in response to specific questions (e.g., Picard et al., 2012) and to remember incidental contextual details (e.g., Sluzenski et al., 2006). While such improvements are most rapid during the preschool and early school years, there continue to be advances well into adolescence (Newcombe et al., 2007). Indeed, in a study that evaluated strategically driven episodic remembering in participants aged 10 to 75 years, Shing et al. (2010) demonstrated gains through to early adulthood. These results can be attributed to the slow development of the prefrontal cortex, which controls intentional retrieval and is one of the last brain regions to reach maturity.

Notwithstanding this body of evidence, another sizeable literature documents the phenomenon of *involuntary memories*, that is, memories that come to mind spontaneously with no deliberate effort at recall. When such memories refer to coherent, contextually bound events (e.g., a person walking past a Greek restaurant suddenly remembers a meal they ate in Corfu while on holiday) they are known as involuntary autobiographical memories (IAMs). IAMs have been studied using a variety of techniques, including diary methods (e.g., Berntsen, 1996) and laboratory vigilance tasks (e.g., Schlagman & Kvavilashvili, 2008), but the findings have been consistent in suggesting that IAMs refer mainly to specific incidents, occur frequently in everyday life, and are often triggered by cues that can be either external (i.e., environmental) or internal (i.e., thought‐related; Berntsen, 2010; Laughland & Kvavilashvili, 2018; Mace, 2004; Rasmussen et al., 2015). Neuroimaging studies show that IAMs rely on the same medial‐temporal and posterior‐parietal brain regions that support voluntary remembering. However, IAMs are distinguished by diminished neural activity in the prefrontal cortex (see Hall et al., 2014). This is indicative of less effortful recall and suggests that such memories are brought to mind via *associative* retrieval mechanisms, rather than the *generative* retrieval processes that characterize voluntary autobiographical memories (Conway, 2005). An important theoretical prediction that emerges from these findings is that age effects on children's recall of IAMs (in terms of both the frequency and the contents of recalled memories) should be less pronounced than age effects in strategically driven episodic remembering (Krøjgaard et al., 2017).

Involuntary memories can also comprise isolated words, images or music, in which case they are referred to as *involuntary semantic memories* or *mind‐pops*, and such fragmentary memories are considered to be different from IAMs. For example, Kvavilashvili and Mandler (2004, Study 4) asked a large sample of adults to keep two separate diaries of mind‐pops and IAMs, recording their content and the context in which they occurred (e.g., triggers, ongoing activities), over two consecutive 1‐week periods (in counterbalanced order). Results showed that while the proportion of participants who had mind‐pops was lower than that usually reported for IAMs, the majority of participants (62%) did record at least one mind‐pop, with some people claiming to experience the phenomenon almost daily (especially in the case of music mind‐pops). Although both IAMs and mind‐pops were reported to have occurred during mundane, relatively effortless activities, mind‐pops were more often reported as coming out of the blue without any obvious cues (e.g., the name “Tom Cruise” or a song by Beatles suddenly coming to mind while doing the washing‐up). However, the occurrence of mind‐pops was not completely random, because in some cases participants were able to identify that the contents of their mind‐pop had been encountered in the recent past (e.g., having a mind‐pop of the phrase “corporal punishment” and later discovering that the phrase had been encountered in work documents reviewed 5 days earlier).

Based on these findings, Kvavilashvili and Mandler (2004) attributed mind‐pops to long‐term conceptual priming within the brain's semantic network. Specifically, they suggested that such briefly encountered information can remain in a heightened state of activation over a prolonged period and thus is prone to burst into conscious awareness suddenly and unexpectedly, especially when the mind is wandering or otherwise unengaged. More recent research examining individual differences in the frequency of mind‐pops has revealed that a heightened propensity for them is linked with greater creativity and openness to experience (Zhang et al., 2016). The limited data on mind‐pops thus indicate that they are not a product of the episodic memory system; rather, these types of involuntary memories are devoid of contextual information and lacking any involvement of self (Kvavilashvili & Mandler, 2004). Moreover, unlike repetitive earworms or “stuck song syndrome” studied in research on involuntary musical imagery (Beaman & Williams, 2010; Liikkanen & Jakubowski, 2020), ordinary mind‐pops in the form of words, images, or music are mostly transient one‐off occurrences that do not disrupt one's ongoing activities.

## IAMs in children

To date, the handful of investigations of involuntary memories in children has focused exclusively on IAMs. Early evidence for the existence of IAMs during the preschool years came from structured diary studies for which parents recorded examples of their children's everyday conversations about memories. Although the focus of such studies was children's ability to recall salient events in response to explicit prompts (e.g., birthday party, family outing), often the parents recorded instances where children referred spontaneously to things that happened to them in the past, especially when cued by related objects or events in the current environment (Nelson & Ross, 1980; Reese, 1999; Todd & Perlmutter, 1980).

With parent‐report methods, though, it is difficult to exclude the possibility that at least some of the memories occurred because the children were in an intentional retrieval mode. To overcome this problem, Krøjgaard et al. (2014) developed a behavioral method to elicit IAMs under controlled laboratory conditions. Specifically, they invited 3.5‐year‐old children to a laboratory setting on two occasions where they witnessed a researcher demonstrate how to operate one of two devices, a “magic shrinking machine” and a “crazy duplicator.” Around 7 months later, each child was brought back to the laboratory and, while waiting alone with their parent after the researcher left the room, was video recorded in conversation with their parent (parents had strict instructions not to raise the subject of the previous laboratory visits). Subsequently, children were left to play with the machines while their behaviors and comments to the researcher were video recorded, before finally being asked explicit questions about how the machines worked. In comparison to control children who had never visited the laboratory before, children in the experimental group produced more utterances relevant to the target events in conversation with their parent, carried out more correct actions while playing with the machines, and gave more correct answers when asked explicit questions about the machines.

Further research replicated these findings using different events (Krøjgaard et al., 2017), even when the props for the original activities were placed in a different room when children returned for testing (Sonne et al., 2019). Despite no effect of age on the frequency of children's spontaneous reports about memories from the previous laboratory visit, older children significantly outperformed younger ones at answering explicit questions about the events (Krøjgaard et al., 2017; see also Martin‐Ordas et al., 2017). Given young children's difficulties with intentional episodic retrieval, it seems likely that IAMs are the predominant method by which they remember their past experiences (Berntsen, 2010, 2012).

## The present study: Development of metamemory for IAMs and mind‐pops

As described above, parent‐report and laboratory studies provide initial support for the idea that IAMs can be experienced by children and that their familiarity with this phenomenon may start from a fairly young age. Therefore, in the present exploratory investigation, we sought for the first time to examine children's awareness and understanding of IAMs, as well as mind‐pops, in their daily lives, that is, their *metamemory* for these phenomena. Flavell and Wellman (1977) distinguished between declarative metamemory, that is, explicit knowledge about how memory operates, and procedural metamemory, that is, largely implicit knowledge about how to regulate memory for optimum performance. Subsequent theorizing identified two interrelated procedural operations, namely, monitoring and control (Nelson & Narens, 1990; Schneider & Lockl, 2002). While *monitoring* refers to introspection about the current state of one's memory (involving feelings of knowing, judgments about ease of learning/retrieval, etc.), *control* involves the application of information gained through monitoring to improving memory performance. Research on the development of metamemory indicates considerable growth during middle childhood (review by Schneider & Löffler, 2016). To the best of our knowledge, though, all such research to date has been limited to children's voluntary memory.

To examine developmental trends in metamemory for IAMs and mind‐pops, we compared the results for four age groups, namely, 5‐, 7‐, and 9‐year‐olds, and young adults. Given reliance on verbal self‐report to gauge children's metamemory in previous research (review by Schneider, 2010), we elicited data via a structured interview with two parts for IAMs and mind‐pops, respectively. In both parts of the interview, we sought to gauge participants' awareness of having IAMs and mind‐pops during everyday life, that is, their monitoring of such phenomena. In particular, we started by providing examples of IAMs and asked participants to reflect on whether IAMs ever happened to them. Those who responded affirmatively were then asked to describe an example of an IAM from their own experience and to estimate the frequency of their IAMs. We next shifted focus to mind‐pops and repeated the procedure, collecting information about word, image, and music mind‐pops in turn. At the end of the interview, we also evaluated participants' explicit understanding of mind‐pops, that is, their declarative metamemory for mind‐pops, by asking them to suggest possible reasons why mind‐pops occur. Unlike IAMs, mind‐pops are often perceived by adults as random occurrences (because of the absence of easily identifiable triggers) and we wanted to see whether any of our participants would suggest either prior exposure (i.e., long‐term priming) or triggers as possible mechanisms underlying the occurrence of their mind‐pops (Kvavilashvili & Mandler, 2004).

The choice of age groups and the interview method was based on the results of extensive piloting of the method with children aged 7–8 (*n* = 20), 9–10 (*n* = 25), 11–13 (*n* = 19), and 14–15 years (*n* = 17) and a group of undergraduate students (*n* = 47). No significant differences between young adults and children aged 9–10 and above were obtained on any of the measures. Although 7‐ to 8‐year‐olds did not differ from adults in terms of their monitoring of IAMs, they were less likely to endorse some of the questions about their experiences of mind‐pops. Given that these young children understood the interview questions and were able to provide meaningful answers to questions about IAMs and mind‐pops, in the present study, in addition to adults and 7‐ and 9‐year‐olds, we included a group of 5‐year‐old children. Because participants needed to engage in effortful retrieval processes to answer questions about their experience of involuntary memories, we predicted that 9‐year‐old‐children and adults would indicate greater awareness of both IAMs and mind‐pops and be better able to supply examples than 7‐ and especially 5‐year‐old‐children. Nevertheless, given that 3‐ to 5‐year‐old‐children have demonstrated the ability to metacognitively monitor various cognitive states (for a review, see Lyons & Ghetti, 2010), and that 30% of 5‐year‐olds were able to describe their spontaneous thoughts in the “no think” task (Flavell et al., 2000; see also Ghetti et al., 2011), we hypothesized that even some of the youngest age group would acknowledge having IAMs and/or mind‐pops and succeed in recalling relevant examples. Likewise, while we expected the self‐reported frequency of involuntary memories to be highest among adults and 9‐year‐olds, we assumed that any children who claimed to experience IAMs and/or mind‐pops must do so sufficiently often for these phenomena to have intruded on their notice. Finally, based on the literature documenting protracted childhood development of declarative metamemory, particularly relating to knowledge of variables influencing memory retrieval, we anticipated that adults would outperform children in their ability to suggest credible mechanisms for mind‐pops.

Additionally, we performed exploratory content analyses of participants' examples of IAMs and mind‐pops to ascertain their familiarity with the phenomena and ability to provide plausible examples. Because previous research has shown that adults find it much easier to identify the presence of incidental triggers for IAMs than mind‐pops (Kvavilashvili & Mandler, 2004), we were interested to see whether the same was true of children. Therefore, IAMs were coded also in terms of whether the IAM example included a description of the trigger eliciting the IAM. To further evaluate the typical content of self‐reported involuntary memories across development, we also scored the emotional valence of reported IAMs and analyzed different types of mind‐pops within each of the three mind‐pop categories (e.g., whether “image” mind‐pops constituted people, places, objects, etc.).

## METHOD

### Participants

A total of 144 participants took part in the study. The child participants were a convenience sample recruited from two primary schools near the University of Hertfordshire in north London (UK), subject to written and informed parental consent and approval from head teachers and our university ethics committee. Because the schools had similar ethnic composition (predominantly White) and socioeconomic background (families with lower‐middle‐ to middle‐class income), it was not deemed necessary to collect information on each child's ethnicity and socioeconomic status. There were 35 five‐year‐olds (*M*_age_ = 67.83 months; range = 60–71 months; 15 males), 37 seven‐year‐olds (*M*_age_ = 90.49 months; range = 84–95 months; 15 males), and 35 nine‐year‐olds (*M*_age_ = 114.17 months; range = 108–119 months; 17 males). The young adult participants were 37 undergraduate students who took part voluntarily and were recruited opportunistically from around the university campus (*M*_age_ = 20.73 years; range = 18–23 years; 11 males). All participants were fluent English speakers, and the children were typically developing. Participants were tested in February–March 2012.

### Materials and procedure

Interviews were conducted individually in a quiet area (e.g., school library, vacant teaching room, or laboratory) by two female researchers who shared data collection for all age groups. Based on extensive piloting on both children and adults, two interview schedules were designed, one suitable for children and the other suitable for adults (see Appendix). They comprised the same pre‐specified questions about involuntary memories but differed slightly in the examples of IAMs and mind‐pops supplied to participants for explanatory purposes. Adults were informed that the aim of the study was to investigate different types of involuntary memory while children were told only that the interviewer wanted to ask them a few questions about their memory. It was made clear that there were no right or wrong answers and that the interview would last around 10 min.

As a warm‐up exercise, participants were asked to comment on whether they thought they had a good memory. Following this, they were questioned about their experiences of IAMs and mind‐pops. In each case, the interviewer began by describing the relevant phenomenon and providing a couple of examples (see Appendix). If participants agreed that such things happened to them too, then they were asked to describe an example from their own experience and to estimate how often they had involuntary memories of this kind on a 5‐point scale (1 = *never*, 2 = *very rarely*, 3 = *occasionally*, 4 = *quite often*, 5 = *every day*). The scale was shown to all participants and the descriptors were also read out to children and paraphrased if necessary (e.g., “hardly ever” and “sometimes”). Participants provided a frequency rating even if they were not able to provide an example of involuntary memory from their everyday life. In the case of mind‐pops, participants were first asked whether they experienced mind‐pops in general. If the participant responded affirmatively, they were then asked about specific types of mind pops (namely, words, images, and music). For each sub‐type, participants had to indicate if they had experienced this particular form of mind pop, and if yes, to provide an example in their own words, and rate the frequency of their occurrence on the same 5‐point rating scale as for IAMs. Finally, participants were invited to speculate on the reasons for having mind‐pops.

## RESULTS

Results are presented in five main sections examining (1) age effects on the experience of IAMs (yes/no) and their frequency, (2) content analysis of IAMs, (3) age effects on the experience of word, image, and music mind‐pops, (4) content analysis of word, image, and music mind‐pops, and (5) age effects on explanations of mind‐pops. Where analyses were applied to contingency tables, we used Fisher–Freeman–Halton Exact tests (FET) to overcome the problem of highly unequal cell sizes (Freeman & Halton, 1951). Findings indicating significant age effects were followed up with *z* tests for the difference between independent proportions.

Due to the exploratory nature of our study, we had no literature on which to base predictions regarding the likely magnitude of age differences in reporting of involuntary memories. Accordingly, for each of the contingency analyses, we conducted retrospective sensitivity analysis using G*Power 3 (Faul et al., 2007) to identify the minimum effect size that could reliably be detected with power (i.e., 1 − *β*) = .80, and *α* = .05, given the known group sizes. Results showed that for 19 of 22 contingency analyses the group sizes were sufficient to detect a medium effect size (range = 0.28 to 0.39), while for the remaining three analyses, all with smaller *n*s, they were sufficient to detect a large effect size (range = 0.45 to 0.50). For follow‐up tests of the difference between proportions, group sizes were sufficient to detect small to medium effects (range = 0.20 to 0.38) in most cases (66%).

### Age effects on the experience of IAMs

In all age groups, the majority of participants claimed to have experienced IAMs, with percentages ranging from 77% in 5‐year‐olds to 97% in adults (see Table 1, panel A). However, there was a positive association between age and whether participants reported ever having experienced an IAM, *p* = .033 (FET). Follow‐up tests revealed that the proportion of participants who stated that they had IAMs was lower for 5‐year‐olds than adults, *z* = −2.54, *p* = .011, and lower for 7‐year‐olds than adults, *z* = −2.47, *p* = .014. No other age comparisons were reliable, *p* > .05.

**TABLE 1:** Numbers (percentages) of participants per age group who claimed to experience IAMs (panel A), provided an IAM example (panel B) and as a function of type of example (IAM with retrieval context, IAM without retrieval context, word pop‐up, other/invalid; panel C)

Age group	Yes	No
(A) Claimed to experience IAMs		
5‐year‐olds (*n* = 35)	27 (77%)	8 (23%)
7‐year‐olds (*n* = 37)	29 (78%)	8 (22%)
9‐year‐olds (*n* = 35)	31 (89%)	4 (11%)
Adults (*n* = 37)	36 (97%)	1 (3%)
(B) Provided example of IAM		
5‐year‐olds (*n* = 27)	18 (67%)	9 (33%)
7‐year‐olds (*n* = 29)	25 (86%)	4 (14%)
9‐year‐olds (*n* = 31)	29 (93.5%)	2 (6.5%)
Adults (*n* = 36)	35 (97%)	1 (3%)

Age group	With context	Without context	Word pop‐up	Other/invalid
(C) Type of IAM example				
5‐year‐olds (*n* = 18)	13 (72%)	3 (17%)	1 (5.5%)	1 (5.5%)
7‐year‐olds (*n* = 25)	22 (88%)	1 (4%)	1 (4%)	1 (4%)
9‐year‐olds (*n* = 29)	23 (79%)	2 (7%)	2 (7%)	2 (7%)
Adults (*n* = 35)	26 (74%)	6 (17%)	1 (3%)	2 (6%)

Abbreviation: IAM, involuntary autobiographical memories.

Based on the data of 123 participants who claimed to have IAMs, we conducted a one‐way ANOVA to compare age groups in terms of their self‐rated frequency of IAMs (1 = *never*; 2 = *very rarely*; 3 = *occasionally*; 4 = *quite often*; 5 = *every day*). Note that none of the participants responded “*never*.” This analysis yielded a significant main effect of age, *F*(3, 119) = 7.56, *p* < .001, *η* ^2^ = .16. Tukey tests showed that frequency ratings were significantly higher for the adults (*M* = 3.89, *SD* = 0.92) than for the 7‐ and 9‐year‐olds (*M* = 2.79, *SD* = 1.08 and *M* = 3.16, *SD* = 0.74, respectively), *p* values < .02, but not for the 5‐year‐olds (*M* = 3.44, *SD* = 1.09), *p* = .27. No significant differences in frequency ratings were found between the three groups of children, *p* > .05.

### Content analysis of IAM descriptions

#### Autobiographical details and retrieval context

Of participants who claimed to experience IAMs (*n* = 123), most provided an example upon request (and none merely paraphrased the exemplars offered earlier by the interviewer). Examples were provided by 18 five‐year‐olds (67%), 25 seven‐year‐olds (86%), 29 nine‐year‐olds (94%), and 35 adults (97%), which represented a significant rise with age, *p* = .003 (FET; see Table 1, panel B). Follow‐up tests revealed that the proportion of participants who gave an example of an IAM was significantly lower for 5‐year‐olds than adults, *z* = −3.23, *p* = .001, but no other age comparisons were reliable, *p* > .05.

The content of these examples was scored according to whether the participant described, first, an autobiographical episode, and second, a retrieval context suggesting that the memory was recalled involuntarily. Specifically, examples were deemed autobiographical if they recounted a memory of a particular event in the participant's life. These examples included unique experiences of having an IAM (e.g., *when I saw an aeroplane, it reminded me of when I went on an aeroplane*), repeated experiences of a certain IAM (*when I see a suitcase, I remember when I was hiding in a suitcase*), or more generic descriptions of types of IAMs (e.g., *if I have a type of food I had on holiday, I remember my holiday*). Despite differences, all these descriptions refer to remembering a specific autobiographical event such as flying on an aeroplane, hiding in a suitcase, or being on holiday. Examples were deemed to include a retrieval context indicative of involuntary retrieval if participants referred to an incidental cue (either external or internal) that triggered the memory or, alternatively, described an unexpected popping of the memory into conscious awareness during unrelated activities. Based on this scoring scheme, examples were coded into four categories by two independent raters who showed high agreement (Cohen's *κ* = .86, *SE* = .057; see Table 1, panel C). Scoring discrepancies between raters were settled by discussion.

The first category (*IAM with context*) comprised descriptions that included both autobiographical details and a retrieval context that specified either a trigger or a popping‐up experience. As can be seen from Table 1 (panel C), the vast majority of IAM descriptions (79%) fell into this category, for example, “*When I see ice, it reminds me of when I went ice‐skating*,” “*Days out with my family pop into my head at school*” (5‐year‐old); “*When I went to McDonalds and saw my friend it reminded me of when I saw her at the park*” (7‐year‐old); “*I remember when I spilt tomato sauce on my shorts when I see the sauce*,” “*Arguments I have had pop into my head after they have happened*” (9‐year old); and “*I saw a ginger cat and it reminded me of when my brother told me he likes Garfield*” (adult).

The second category (*IAM without context*) comprised autobiographical descriptions for which participants made no mention of where or how the memory was recalled. That is, the spontaneous or involuntary nature of retrieval was not explicitly stated. Examples of this type were reported on relatively few occasions (11%), and included, “*I remember when I went to the London Transport Museum*” (5‐year‐old); “*When my sister was born—she is 5 years old now*” (7‐year‐old); “*I remember when my grandad died*” (9‐year‐old); and “*My holiday in Florida few years ago*” (adult).

Two further categories comprised descriptions that did not qualify as IAMs. The first of these (*word pop‐up*) referred to an isolated word or name breaking unexpectedly into the participant's consciousness. Such descriptions were rare and in most cases, the participants reported these experiences as arising following deliberate but unsuccessful retrieval attempts, for example, “*My friend asked me what I was reading the other day, and at the time I couldn't remember but it popped into my head later on*” (5‐year old); “*In drama I forgot my line, but as I was acting out I remembered from when we practiced*” (7‐year old); and “*Sometimes I can't think of the name of someone I've just met but it will come back to me later on*” (adult). The final category (*invalid/other*) comprised only a small number of descriptions referring to some other idiosyncratic experiences (e.g., keeping a diary) or odd phenomena (e.g., Déjà vu).

**FIGURE 1:**
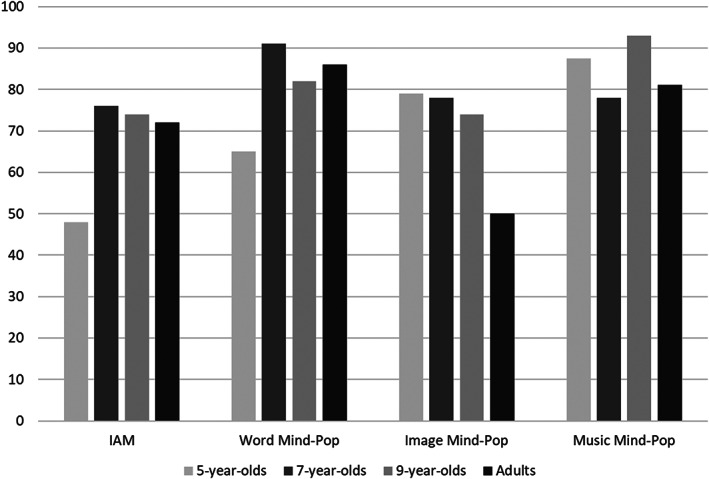
Of participants in each age group who claimed to experience IAMs and different types of mind‐pops, the percentage who gave a valid example. IAM, involuntary autobiographical memories.

As shown in Table 1 (panel C), among participants who offered an example of an IAM the percentages of descriptions falling into these four categories were similar across age groups. This impression was confirmed by a contingency analysis showing no significant association between age group and type of example, *p* = .857 (FET). To assess age effects by a more stringent test, we compared the proportions of participants who either did or did not provide a clear example of an IAM (i.e., an IAM including the retrieval context), out of all participants in each age group who claimed to experience IAMs (see Figure 1). Although the likelihood of describing an IAM with retrieval context was lower in 5‐year‐olds than in other age groups, this difference was not statistically significant, *p* = .104 (FET).

#### Triggers and emotional valence

Research on IAMs in adults, using both diary and laboratory methods, has consistently demonstrated the importance of incidental cues in triggering IAMs (Berntsen, 1996; Mace, 2004; Mazzoni et al., 2014; Schlagman & Kvavilashvili, 2008). In addition, in terms of emotional valence, IAMs tend to be neutral or positive rather than negative (Berntsen, 1996; Schlagman & Kvavilashvili, 2008). We, therefore, conducted two further content analyses to see whether there were any age effects on the likelihood that participants mentioned triggers in their descriptions of IAMs and whether the IAM descriptions were predominantly of neutral and/or positive events.

In the first analysis, descriptions classed as IAMs with context were coded by two independent raters into two categories: (1) those that identified an incidental external or internal cue that was responsible, according to the participant, for triggering the memory, and (2) those that failed to mention any trigger (thus, the IAM was an unexplained pop‐up). Examples mentioning a trigger included, “*I might see something and it would remind me of what I did at school*” (5‐year‐old); “*When I see something in a film that I've done, I remember it*” (7‐year‐old); “*I saw a cloud shaped as a teddy and this reminded me of when I got my teddy*” (9‐year‐old); and “*I smelt a smell like my gran's flat and I remembered when I got lost in her building when I was little*” (adult). Examples lacking a reported trigger included, “*Days out with my family pop into my head at school*” (5‐year‐old); “*When I'm at home watching TV I remember what I did at school*” (7‐year‐old); “*I was talking to my friend and then just remembered what I had for dinner*” (9‐year‐old); and “*I would be walking down the street and I'll remember where I went on holiday*” (adult). The agreement between the coders was excellent (*κ* = .94, *SE* = .04) and two cases of disagreement were settled by discussion. As shown in Table 2 (panel A), most accounts referenced a trigger to the IAM (75%), and the association between age and the frequency with which a trigger was reported was not significant, *p* = .115 (FET).

**TABLE 2:** Numbers (percentages) of participants per age group, who described an IAM with retrieval context, as a function of reporting a trigger (yes, no; panel A) and memory valence (positive/neutral, negative, unclear; panel B)

Age group	Yes	No
(A) Trigger		
5‐year‐olds (*n* = 13)	11 (85%)	2 (15%)
7‐year‐olds (*n* = 22)	12 (55%)	10 (45%)
9‐year‐olds (*n* = 23)	19 (83%)	4 (17%)
Adults (*n* = 26)	21 (81%)	5 (3%)

Age group	Positive/neutral	Negative	Unclear
(B) IAM valence			
5‐year‐olds (*n* = 13)	10 (77%)	1 (7.5%)	2 (15.5%)
7‐year‐olds (*n* = 22)	14 (63.5%)	3 (13.5%)	5 (23%)
9‐year‐olds (*n* = 23)	19 (83%)	3 (13%)	1 (4%)
Adults (*n* = 26)	22 (85%)	2 (7.5%)	2 (7.5%)

Abbreviation: IAM, involuntary autobiographical memories.

The second content analysis was similarly based on the pool of IAMs with context. Two independent raters coded these memory descriptions according to whether they referred to generally pleasant or neutral events (e.g., holidays, activities with family or friends, innocuous events), negative events (e.g., injuries or illness, arguments, bereavement, nightmares) or were unclear (i.e., it was not possible to ascertain the valence of the event, e.g., “*When I'm in the car, memories pop into my head when we go past somewhere*” (5‐year‐old). The agreement between the coders was excellent (*κ* = .91, *SE* = .053), and disagreements were settled by discussion. As indicated by Table 2 (panel B), the bulk of memories fell into positive/neutral category (77%) and distributions did not differ according to age, *p* = .533 (FET).

In summary, the results showed that most participants in all age groups (>75%) reported that they experienced IAMs, and did so fairly often, but in line with predictions, adults were more likely than 5‐ and 7‐year‐old‐children to report experiencing IAMs, and their self‐rated frequency of IAMs was higher than in 7‐ and 9‐year‐olds (albeit not 5‐year‐olds). Importantly, although adults were more likely than 5‐year‐olds to recall an IAM example, there was no significant effect of age on the ability to produce a clear example, or in the types of examples reported, with participants in all age groups tending to describe not just their memory but the circumstances surrounding its retrieval. Moreover, a majority of participants in every age group reported a trigger for their IAM and in most cases the IAM was of an emotionally positive or neutral nature.

### Age effects on the experience of mind‐pops

The first column of Table 3 shows numbers (and percentages) of participants in each age group who claimed to experience mind‐pops of any kind. As with IAMs, most participants said they had mind‐pops and there was a significant rise in the percentage of participants across age groups who answered the question affirmatively, from 71% in 5‐year‐olds to 94% and 97% in 9‐year‐olds and adults, respectively, *p* = .006 (FET). Follow‐up tests revealed that the proportion of 5‐year‐olds who reported experiencing mind‐pops was lower than in 9‐year‐olds (*z* = −2.53, *p* = .011) and in adults (*z* = −3.04, *p* = .002). No other age comparisons were reliable, *p* > .05.

**TABLE 3:** Numbers (percentages) of participants per age group who claimed to experience mind‐pops of any kind, and of these, numbers (and percentages) of participants who claimed to experience word, image, and music mind‐pops

Age group	Claimed to experience mind‐pops			
	Any	Word	Image	Music
5‐year‐olds (*n* = 35)	25 (71%)	17 (68%)	14 (56%)	16 (64%)
7‐year‐olds (*n* = 37)	32 (86%)	22 (69%)	23 (72%)	27 (84%)
9‐year‐olds (*n* = 35)	33 (94%)	22 (67%)	23 (70%)	27 (82%)
Adults (*n* = 37)	36 (97%)	21 (58%)	18 (50%)	36 (100%)

*Note*: Percentages for word, image, and music mind‐pops are based on the number of participants in each age group who claimed to experience mind‐pops in general (irrespective of type).

Considering only participants who claimed to experience mind‐pops in general (*n* = 126), further analyses were conducted to examine the association between age and the self‐reported experience of different types of mind‐pops, that is, words, images, and music (see columns 2–4 in Table 3). No significant age effects were evident for either words, *p* = .811 (FET), or images, *p* = .202 (FET). However, there was a developmental increase in the reported experience of mind‐pops involving music, *p* = .001 (FET). Tests of the difference between proportions showed an equivalent experience of music mind‐pops between the 5‐, 7‐, and 9‐year‐olds, *p* values >.05, but a significantly higher number of such reports among adults compared to 5‐year‐olds, *z* = 3.90, *p* < .001, 7‐year‐olds, *z* = 2.50, *p* = .012, and 9‐year‐olds, *z* = 2.66, *p* = .008.

Among participants who claimed to experience a particular type of mind‐pop (word, image, and/or music), the self‐reported frequency ratings were compared between the four age groups using one‐way ANOVAs (1 = *never*; 2 = *very rarely*; 3 = *occasionally*; 4 = *quite often*; 5 = *every day*). Note that none of the participants responded “*never*” for any type of mind‐pop. There was no effect of age on frequency ratings for image mind‐pops (5‐year‐olds *M* = 3.57, *SD* = 1.02; 7‐year‐olds *M* = 3.52, *SD* = 1.08; 9‐year‐olds *M* = 3.57, *SD* = 0.99; adults *M* = 3.50, *SD* = 1.04), *F*(3, 74) = 0.02, *p* = .996, *η* ^2^ = .00), or music mind‐pops, (5‐year‐olds *M* = 3.50, *SD* = 1.03; 7‐year‐olds *M* = 3.59, *SD* = 1.01; 9‐year‐olds *M* = 3.48, *SD* = 0.85; adults *M* = 3.83, *SD* = 1.06), *F*(3, 102) = 0.81, *p* = .491, *η* ^2^ = .02. However, there was a significant outcome for word mind‐pops, (5‐year‐olds *M* = 3.88, *SD* = 0.93; 7‐year‐olds *M* = 3.09, *SD* = 1.15; 9‐year‐olds *M* = 2.95, *SD* = 0.90; adults *M* = 3.52, *SD* = 0.98), *F*(3, 78) = 3.46, *p* = .020, *η* ^2^ = .12, with Tukey tests indicating a higher frequency among 5‐year‐olds than 9‐year‐olds only, *p* = .026.

### Content analysis of mind‐pops

Participants' examples of word, image, and music mind‐pops were evaluated by two independent raters. Unlike IAMs, relatively few participants described any retrieval context for their memories, instead simply reporting what it was that they remembered. Accordingly, the coding of each type of mind‐pop was intended to distinguish between different types of semantic content and to exclude examples that did not appear to be mind‐pops.

#### Content analysis of word mind‐pops

Of participants who claimed to experience word mind‐pops, most provided an example upon request (none of the descriptions repeated or paraphrased an example offered earlier by the interviewer). Examples were provided by 15 five‐year‐olds (88%), 21 seven‐year‐olds (95%), 19 nine‐year‐olds (86%), and 20 adults (95%), with results failing to differ significantly by age, *p* = .643 (FET). Examples were coded by the raters into the following four categories: (1) proper names, (2) common nouns, (3) tip‐of‐the‐tongue experiences, and (4) invalid/other examples. Tip‐of‐the‐tongue experiences were treated as a separate category from word mind‐pops because they are regarded as a distinct phenomenon in the literature, being always preceded by unsuccessful retrieval attempts (Reason & Lucas, 1984), while word mind‐pops come to mind without any prior attempts to recall them (Kvavilashvili & Mandler, 2004). Examples in the invalid/other category were those that appeared to denote thinking or planning rather than involuntary memory or were otherwise unclassifiable. Agreement on the coding of contents for word mind‐pops was excellent (*κ* = .93, *SE* = .04) and disagreements were settled by discussion.

In the “proper names” category, some examples provided by participants included, “*Friend's name, Anna*” (5‐year‐old); “*Mia ‐it's my sister's friend's name, my friend's name and my cousin's name*” (7‐year‐old); “*Ben (dog), when I was just talking to my friend*” (9‐year‐old); and “*I always think of different people's names*” (adult). In the “common nouns” category, examples included, “*Words that I've heard but don't know what they mean*” (5‐year‐old); “*coach*” (7‐year‐old); “*epiphany*” (9‐year‐old); and “*the word* ‘*expansive*’ *has popped into my head before and I'm not sure why!*” (adult). In the “tip‐of‐the‐tongue” category, examples included, “*I was trying to remember a friend's name for my birthday—I couldn't remember at the time but later it popped into my head*” (5‐year‐old); and “*Sometimes I forget a name and then it later pops into my head*” (5‐year‐old). Finally, “invalid/other” examples included “*The name Luke. I was thinking about what to call my teddy and children if I have them and ‘Luke’ popped into my head*” (9‐year‐old); and “*I usually think of the people that I need to see later in the day, such as tutors/friends*” (adult).

Table 4 shows frequencies (and percentages) of participants in each age group who contributed examples to each of the above four categories. As can be seen, descriptions fell predominantly into the proper names category. While 5‐year‐olds were the only age group to report tip‐of‐the‐tongue experiences, there was no significant effect of age on the distribution of responses, *p* = .081 (FET). Furthermore, taking account of all participants in each age group who claimed to experience word mind‐pops, there was no significant effect of age on the probability that participants proceeded to describe a clear example of a word mind‐pop relating to either a proper name or a common noun, *p* = .227 (FET; see Figure 1).

**TABLE 4:** Numbers (percentages) of participants per age group who provided an example of an experienced word mind‐pop, as a function of type of example (proper name, common noun, tip‐of‐the tongue experience, other/invalid)

Age group	Type of word mind‐pop example			
	Proper name	Common noun	Tip‐of‐the tongue	Other/invalid
5‐year‐olds (*n* = 15)	7 (47%)	4 (26.5%)	4 (26.5%)	0
7‐year‐olds (*n* = 21)	15 (71%)	5 (24%)	0	1 (5%)
9‐year‐olds (*n* = 19)	13 (68.5%)	5 (26.5%)	0	1 (5%)
Adults (*n* = 20)	10 (50%)	8 (40%)	0	2 (10%)

#### Content analysis of image mind‐pops

Of participants who claimed to experience image mind‐pops, and after excluding three instances where the participant repeated the interviewer's example (images of school, university), examples of image mind‐pops were offered by 11 five‐year‐olds (79%), 22 seven‐year‐olds (96%), 19 nine‐year‐olds (83%), and 11 adults (61%). A significant effect of age, *p* = .045 (FET), reflected the fact that 7‐year‐olds were more likely to offer an example of an image mind‐pop than adults, *z* = 2.81, *p* = .005. Examples were coded by the raters into the following four categories: (1) people, pets, or cartoon characters (2) places, (3) objects, and (4) other/invalid examples. Agreement on the coding of contents for image mind‐pops was good (*κ* = .86, *SE* = .05) and disagreements were settled by discussion.

Examples of “people, pets, and characters” included “*Sponge bob*” (5‐year‐old); “*Selena Gomez*” (7‐year‐old); “*my gerbil*” (9‐year‐old); and “*friend*” (adult). Examples of “places” included “*the park*” (5‐year‐old); “*the staff room at school*” (7‐year‐old); “*Cyprus, which is where I come from*” (9‐year‐old); and “*the image of my room at home*” (adult). Examples of “objects” included “*trees*” (5‐year‐old); “*I have the image of my front door sometimes*” (7‐year‐old); “*images of books*” (9‐year‐old); and “*the beach*” (adult). “Other/invalid” *examples* were non‐specific images, (e.g., “*favourite TV programme*” and “*images of football*”), bizarre images (e.g., “*Once I had an image of a hotel with a school on top of it*”) or clearly not an image mind‐pop (e.g., “*I have images of the dreams I have had*” and “*When I am drawing, I picture something to draw*”).

Table 5 shows the number (and percentage) of participants per age group who reported examples in each of these categories. There was no relation between age and the distribution of image mind‐pop examples, *p* = .302 (FET). Taking account of all participants in each age group who claimed to experience image mind‐pops, neither was there a significant effect of age on the probability that participants described a clear example of an image mind‐pop (i.e., an image of a person/pet, a place or an object), *p* = .212 (FET; see Figure 1).

**TABLE 5:** Numbers (percentages) of participants per age group who provided an example of an experienced image mind‐pop, as a function of type of example (person/pet/cartoon character, place, object, other/invalid)

Age group	Type of image mind‐pop example			
	Person/pet/character	Place	Object	Other/invalid
5‐year‐olds (*n* = 11)	5 (45.5%)	1 (9%)	5 (45.5%)	0
7‐year‐olds (*n* = 22)	11 (50%)	3 (14%)	4 (18%)	4 (18%)
9‐year‐olds (*n* = 19)	9 (47%)	3 (16%)	5 (26%)	2 (11%)
Adults (*n* = 11)	2 (18%)	5 (46%)	2 (18%)	2 (18%)

#### Content analysis of music mind‐pops

Of participants who claimed to experience music mind‐pops, and after excluding one instance where the participant repeated the interviewer's example (*Happy Birthday*), examples of music mind‐pops were supplied by 14 five‐year‐olds (88%), 23 seven‐year‐olds (85%), 25 nine‐year‐olds (93%), and 29 adults (81%), with results failing to differ significantly by age, *p* = .651 (FET). Descriptions of music mind‐pops were coded by the raters into three categories: (1) songs, (2) melodies, and (3) invalid/other examples. Agreement between the two raters was lower than with coding other variables, but acceptable (*κ* = .60, *SE* = .16; McHugh, 2012). All five cases of disagreement, out of 91 examples, were settled by discussion.

The vast majority of music mind‐pop examples (ranging from 93% in adults to 100% in 5‐year‐olds) fell into the “songs” category, for example, “*A song we learnt in reception pops into my head sometimes*” (5‐year‐old); “*Mamma Mia*” (7‐year‐old); “*When I'm going to sleep, Tiny Tempah*” (9‐year‐old); and “*Songs that are appropriate to a certain situation pop into my head*” (adult). Examples that referred to “melodies” included, “*If I hear my brother playing the flute during the day, I will hum the tune*” (7‐year‐old); and “*Match of the day tune*” (9‐year‐old). There were only two examples classed as “invalid/other.” A description by one 7‐year‐old (“*When I am alone in the playground a song pops in my head that I make up as I go along*”) was deemed to reflect a creative process rather than a music mind‐pop. Another 7‐year‐old's description (“*If I only hear the first part of a song then the words of the rest of the song pop into my head*”) was deemed to refer to words popping into mind rather than the song itself.

Table 6 shows the number (and percentage) of participants per age group who reported examples in each of the three categories. There was no relation between age and the distribution of music mind‐pop examples, *p* = .569 (FET). In addition, when taking account of all participants in each age group who claimed to experience music mind‐pops, there was no significant effect of age on the proportion of participants who provided a clear example of a music mind‐pop (i.e., a song or a melody), *p* = .456 (FET; see Figure 1).

**TABLE 6:** Numbers (percentages) of participants per age group who provided an example of an experienced music mind‐pop, as a function of type of example (song, melody, other/invalid)

Age group	Type of music mind‐pop example		
	Song	Melody	Other/invalid
5‐year‐olds (*n* = 14)	14 (100%)	0 (0%)	0 (0%)
7‐year‐olds (*n* = 23)	20 (87%)	1 (4%)	2 (9%)
9‐year‐olds (*n* = 25)	24 (96%)	1 (4%)	0 (0%)
Adults (*n* = 29)	27 (93%)	2 (7%)	0 (0%)

In summary, results for mind‐pops showed that most participants in all age groups claimed to have mind‐pops in general (>70%), but adults and 9‐year‐old‐children were more likely to claim familiarity with mind‐pops than 5‐year‐olds. In terms of experiencing specific types of mind‐pops, there was an age‐related rise only for music mind‐pops with adults being more likely to claim having them than children in all three age groups. However, no differences were found between adults and children for experiencing word and image mind‐pops. Similarly, no age effects were apparent for reported frequencies of mind‐pops and the ability to supply an example of a mind‐pop in any category (word, image, or music), or to provide a clear example of word, image, and music mind‐pops (see Figure 1). Regardless of age, proper names were given more frequently than common nouns as examples of word mind‐pops, and songs were given more frequently than melodies as examples of music mind‐pops.

### Age effects on explanations of mind‐pops

All participants who claimed to experience mind‐pops, regardless of whether they supplied examples, were asked why they thought such phenomena occurred. Explanations were offered by 5 five‐year‐olds (20%), 22 seven‐year‐olds (69%), 24 nine‐year‐olds (73%), and 34 adults (94%), which represented a significant increase with age, *p* < .001 (FET). Follow‐up tests of the difference between proportions were significant for all age comparisons, *p* < .05, except between 7‐ and 9‐year‐olds. Responses were coded into the following five categories: (1) previous encounter (i.e., mind‐pops are primed), (2) current cue (i.e., mind‐pops are triggered by something in the immediate environment or, alternatively, by one's present thoughts, mood, or emotion), (3) appeal (i.e., mind‐pops represent things that are liked), (4) multiple reasons, and (5) other responses (see Table 7).

**TABLE 7:** Numbers (percentages) of participants per age group as a function of explanation for mind‐pop occurrence (recent encounter, current cue, liking/appeal, multiple reasons, other)

Age group	Recent encounter	Current cue	Liking/appeal	Multiple reasons	Other
5‐year‐olds (*n* = 5)	3 (60%)	1 (20%)	0	0	1 (20%)
7‐year‐olds (*n* = 22)	9 (41%)	2 (9%)	5 (23%)	1 (4%)	5 (23%)
9‐year‐olds (*n* = 24)	15 (63%)	7 (29%)	1 (4%)	0	1 (4%)
Adults (*n* = 34)	11 (32%)	10 (29%)	1 (3%)	6 (18%)	6 (18%)

Examples of *recent encounter* explanations included, “*Because I see my friends a lot*” (5‐year‐old); “*Because I might have heard the songs during the day*” (7‐year‐old); “*Because I might think of them at night and they will pop into my head the next day*” (9‐year‐old); and “*If I've been trying to think of something, the subconscious takes over later on causing it to pop into my head*” (adult). Examples of *current cue* explanations included, “*Mainly at parties—music causes it*” (5‐year‐old); “*Because a word might be said that will make me think of a song*” (7‐year‐old); “Something might remind me of something” (9‐year‐old); and “*Can be associated to emotions at the time*” (adult). Examples of *appeal* explanations included, “*Because I like football so they pop into my head*” (7‐year‐old); “*Because I like them*” (9‐year‐old); and “*I like music a lot*” (adult). Finally, examples of *other* explanations included, “The songs might be because they are catchy” (5‐year‐old); “*My brain makes me do it*” (7‐year‐old); “*Because I'm bored*” (9‐year‐old); and “*When I am not paying full attention*” (adult).

There was a significant effect of age on the prevalence of different explanations for mind‐pops, *p* = .027 (FET), reflecting growing awareness of recent encounters (priming) and current cues (triggers) with development. Considering all participants who claimed to experience mind‐pops, a further analysis was conducted to explore the relation between age and the likelihood that participants identified primes and/or triggers as causing mind‐pops (i.e., combining reports of recent encounters and current cues). For the purposes of this analysis, any participant who mentioned either priming or triggers among multiple reasons was included. In total, primes/triggers were cited by 4 five‐year‐olds (16%), 11 seven‐year‐olds (34%), 22 nine‐year‐olds (67%), and 27 adults (75%), which represented a significant increase with age, *p* < .001 (FET). Such explanations were significantly less frequent for 5‐year‐olds than 9‐year‐olds (*z* = −3.84, *p* < .001) or adults, *z* = −4.53, *p* < .001, and significantly less frequent for 7‐year‐olds than 9‐year‐olds (*z* = −2.60, *p* = .009) or adults, *z* = −3.37, *p* < .001. However, results failed to differ between 5‐ and 7‐year‐olds or between 9‐year‐olds and adults, *p* values > .05.

In summary, findings regarding explanations of mind‐pops were as predicted. There was a significant age‐related rise in the likelihood that participants offered any sort of explanation at all, with most 5‐year‐olds declining to answer, and a developmental shift from citing reasons such as “liking” to demonstrating understanding of recent primes and current triggers as possible causes of mind‐pops.

## DISCUSSION

The phenomenon of involuntary memories has been studied extensively in adults but has received scant attention in children. To date, evidence that children experience involuntary memories is limited to IAMs and has been derived from either parent reports (e.g., Todd & Perlmutter, 1980) or laboratory investigations for which the existence of such memories is inferred from children's spontaneous remarks about a previously staged event when returned to the same location (Krøjgaard et al., 2014, 2017). No previous research has studied the experience of involuntary semantic memories or mind‐pops in children, with only a handful of published studies on this phenomenon in adult population (Elua et al., 2012, 2015; Kvavilashvili & Mandler, 2004; Liu et al., 2019; Zhang et al., 2016).

The goal of the present study was to address these gaps in the literature by taking the novel approach of asking participants directly about their IAMs and mind‐pops and comparing findings for 5‐, 7‐, and 9‐year‐old‐children and young adults. Specifically, after providing age‐appropriate examples of the phenomena of interest, we interviewed participants to ascertain whether they assented to having experienced involuntary memories themselves, how often they thought this happened to them, and their ability to provide examples from their own experience. Given that involuntary memories come to mind effortlessly via associative mechanisms (Berntsen, 2012; Kvavilashvili & Mandler, 2004; Mace, 2010; Schlagman & Kvavilashvili, 2008), and that age effects tend to be attenuated or absent in tasks that rely on automatic retrieval processes, we predicted that many participants in all age groups would claim to have IAMs and mind‐pops. However, we also assumed that the frequency of such claims, as well as the ability to describe specific examples from personal experience, would increase with age due to improvements in the ability to bring relevant instances to mind via strategic retrieval processes.

**FIGURE 2:**
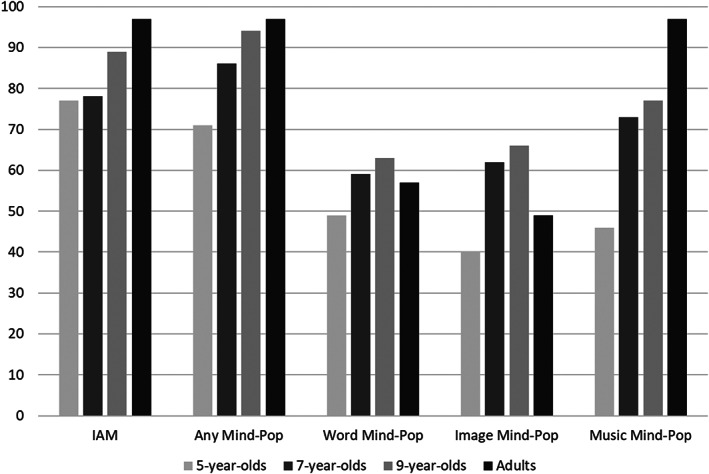
The percentage of participants in each age group who claimed to experience IAMs and different types of mind‐pops. IAM, involuntary autobiographical memories.

These hypotheses were confirmed. Despite a developmental rise in the number of participants who responded affirmatively to the questions regarding whether they ever experienced IAMs and mind‐pops, the vast majority in each age group did so. Thus, 77% of 5‐year‐olds asserted that they had IAMs (rising to 97% in adults) and 71% of 5‐year‐olds asserted that they had mind‐pops in general (again rising to 97% in adults). For illustrative purposes, these high levels of familiarity for IAMs and mind‐pops of any kind, as well as for different types of mind‐pops, are depicted in Figure 2 (the percentages are based on total samples of 5‐, 7‐, and 9‐year‐old children and adults). While fewer participants were able to provide examples of their involuntary memories, particularly the youngest children, a substantial number managed to do so. Moreover, in participants who claimed to experience IAMs, or word, image, and music mind‐pops, statistically significant age effects were not obtained even when examining the likelihood of providing a clear (i.e., valid) example of either an IAM or a word, image, and music mind‐pop, respectively (see Figure 1). Finally, of participants who claimed to experience IAMs, there was no effect of age on the probability that they described an example referring to the involuntary nature of retrieval, that is, noting either a trigger for the memory or a popping‐up experience. This finding suggests that, similarly to adults, having an IAM may be noteworthy for children, prompting them to reflect on and then encode the retrieval experience itself.

Our findings regarding children's self‐reported IAMs highlight the ubiquity of IAMs in their daily lives and represent valuable new evidence that significantly extends the behavioral data obtained in laboratory studies (Krøjgaard et al., 2014, 2017). They further support the proposal that spontaneous retrieval may be a basic mode in which cognition prefers to operate in everyday life, and could be considered a precursor of strategic recall both phylogenetically and ontogenetically (Berntsen, 2010, 2012). The fact that even 5‐year‐olds were cognisant of having IAMs, and in many cases could describe when an IAM happened to them, suggests that children of this age are subject to such phenomena relatively often (i.e., often enough to have mentally registered this type of experience). This conclusion is bolstered by the frequency ratings, which showed an average estimate in each age group falling between “occasionally” and “quite often.” Interestingly, the content analysis of IAMs revealed very similar results across the four age groups. First, in all age groups, most participants reported an identifiable trigger for their IAM. This finding is consistent with previous studies testing adults showing frequent awareness of triggers preceding IAMs (e.g., Berntsen, 1996; Mace, 2004; Schlagman & Kvavilashvili, 2008). Second, almost everyone described an IAM that had a positive or neutral emotional valence. Likewise, a dearth of emotionally unpleasant IAMs has been reported in research with adult participants (e.g., Schlagman & Kvavilashvili, 2008).

Analyses of the self‐reported frequency and content of mind‐pops similarly revealed much developmental invariance. Regardless of age, mind‐pops were judged on average to occur from “occasionally” to “quite often,” and unlike the case for IAMs, participants rarely reported a retrieval context for their mind‐pop examples. Again, findings are consistent with previous research with adult participants because such research has shown that immediate triggers for mind‐pops are relatively difficult to identify (Elua et al., 2015; Kvavilashvili & Mandler, 2004). In line with our content analysis of IAMs, we observed very similar patterns of findings across age groups in the kinds of word, image, and music mind‐pops that were reported. Specifically, participants of all ages were prone to report proper names rather than common nouns for their word mind‐pop examples, and songs rather than melodies for their music mind‐pop examples, but showed no propensity for any particular type of image mind‐pop.

Additionally, we solicited participants' views regarding the possible causes of mind‐pops, with results showing marked age‐related changes in participants' accounts. Unsurprisingly, most 5‐year‐olds had no rational explanation of mind‐pops, generally responding that they had no idea what caused them. Several 7‐year‐olds, however, had apparently notched up sufficient experience of such memories to infer that something must be making them salient; thus, they attributed mind‐pops to the appeal of their content. For both 9‐year‐olds and adults, the focus shifted to current cues (both external and internal) and recent encounters (i.e., priming). Nevertheless, even in the adult age group only 29% of those participants who claimed to experience mind‐pops suggested that mind‐pops might be triggered by current cues. This finding supports those of Kvavilashvili and Mandler (2004) and is congruent with the evidence, reviewed above, that few participants mentioned any kind of retrieval context when describing examples of their own mind‐pops.

Although it might be queried whether the examples of IAMs and mind‐pops provided by the youngest children were genuine, given the active imaginations of most 5‐year‐olds, we argue that their examples should be taken seriously for the following three reasons. First, we discounted any examples that appeared to be modeled closely on those provided beforehand by the interviewer. Indeed, it was striking that only four of the examples from the entire sample, all mind‐pop examples, had to be eliminated on these grounds. Instead, we obtained a varied set of descriptions from all age groups that were notable for their idiosyncrasy. Second, at least in the case of IAMs, many 5‐year‐olds (and children in general) were able to give a detailed example situated within a specific retrieval context that was recounted both in the past‐tense and from their own point of view. Given that this was not the structure of the IAM examples presented by the interviewer, which took the form of suggestions using the words, “You might see [X] and this might remind you of [Y]”, children thus spontaneously framed their responses in language indicative of first‐hand episodic retrieval. Third, as mentioned above, our content analysis of IAMs and mind‐pops uncovered many commonalities between the four age groups. Notably, participants in all age groups were more likely to report names than nouns/verbs as examples of word mind‐pops despite the interviewer offering an example of a noun. Likewise, although both the interviewer's examples of mind‐pops included an irrelevant retrieval context, the trend in all age groups was for participants to mention only the content of their mind‐pop. Overall, then, the content analyses support the conclusion that the reports of involuntary memories were genuine.

In addition to contributing new knowledge to the literature on children's involuntary memories in everyday life, the results of the present study have important implications for developmental research on metamemory. For example, our findings suggest that the concept of “monitoring,” which until now has been investigated mainly in terms of judgments of learning, feelings of knowing, and confidence during learning situations, can be extended to awareness of involuntary memories. In the case of 5‐year‐olds, our data accord with reports that despite age‐related improvements, even very young children show good monitoring abilities for phenomena with which they are well acquainted (review by Schneider & Löffler, 2016). Evidence for impressive monitoring comes from our observation that 77% of 5‐year‐olds claimed to experience IAMs, two‐thirds of these went on to provide a plausible example, and their ratings of the frequency with which IAMs happened to them were at least as high as for the older age groups. Moreover, while we did not ask children directly to reflect on the causes of IAMs, the fact that so many of them spontaneously reported a trigger when giving an example of an IAM suggests that frequent monitoring of IAMs may have helped them to develop declarative knowledge about reminder events.

On the other hand, some of our findings support the conclusion that younger children generally perform worse than older children and adults on tests of monitoring and declarative metamemory (Schneider, 2010). For example, in terms of monitoring, frequency ratings for IAMs among the 5‐year‐olds were equivalent to those for adults, while the frequency ratings provided by 7‐ and 9‐year‐olds were lower than for adults. One possible explanation is that younger children were over‐confident in their memory abilities. It is well documented that 5‐year‐olds give optimistic predictions of their memory encoding and retrieval, an effect that appears to reflect motivational factors rather than metacognitive ones (i.e., children simply prefer to think of themselves as having a good rather than bad memory; Schneider & Löffler, 2016). In addition, in terms of declarative metamemory, the vast majority of 5‐year‐olds were stumped when asked to explain mind‐pops, being unable to offer any plausible account at all, in contrast to older age groups. As already discussed, the developmental rise in the number of participants who suggested that mind‐pops might be caused by triggers fits with the proposal that mind‐pops are driven mainly by long‐term conceptual priming, making the cue event much harder to recognize than is the case for IAMs (Kvavilashvili & Mandler, 2004).

Taken together, the results of the present study suggest several directions for future research. First, it will be important to confirm the apparent discrepancy in young children's declarative knowledge about the contribution of triggers to IAMs versus mind‐pops by interviewing participants explicitly about the causes of both kinds of phenomena. Following on from this, it would make sense to explore the extent to which children's growing appreciation of the role of triggers in IAMs translates into deliberate use of reminders in the aid of voluntary memory retrieval, for example, remembering to carry out future intentions (for a discussion of the important role of reminders in children's prospective memory, see Ryder et al., in press). Given the well‐documented developmental lag between children's cognizance of mnemonic strategies and their ability to put such strategies to effective use (Schneider & Löffler, 2016), it is of interest to see whether children who become aware of triggers through frequent first‐hand experience of IAMs are more likely than other children to learn to apply reminders at a young age.

Another important line of research would be to evaluate children's reports about involuntary memories in relation to their theory of mind (ToM), that is, their comprehension of both their own and other people's mental states. It has been suggested that ToM underpins children's understanding that episodic memories are mental representations of events they experienced in the past (e.g., Perner et al., 2007), as well as giving insight into inferential and evaluative processes (Sodian, 2005). In a longitudinal investigation, Lockl and Schneider (2007) found that early ToM skills predicted children's subsequent knowledge about variables that influence memory (e.g., study time), as well as strategies for optimizing retrospective and prospective memory (e.g., finding a lost possession, remembering to take a pretzel to kindergarten for lunch tomorrow). Although this study focused solely on declarative metamemory, Lockl and Schneider (2007) highlighted the need to examine whether ToM also predicts monitoring processes. In our opinion, the study of involuntary memories provides an ideal vehicle for addressing this issue; for example, future research could probe whether early ToM capabilities predict later individual differences in the ages at which children become aware of having IAMs, and the role of triggers in their IAMs. It is also of interest to see whether, compared to IAMs, more advanced levels of ToM are required before children notice triggers for mind‐pops, given that triggers for IAMs are usually identifiable in the external environment, whereas triggers for mind‐pops more often take the form of internal cues.

Finally, follow‐up studies should address some limitations of the present research. One important weakness is the small number of participants in some of our subsidiary analyses. For example, data presented in Table 5 suggest that adults were more likely to report images of places when providing an example of an image mind‐pop while children were more likely to report images of persons/pets/cartoon characters, but the age difference was not statistically significant. There is therefore a need to test larger samples to detect small but real effects that could have been present in our dataset. Additionally, frequency ratings of IAMs and mind‐pops were obtained from all participants who claimed to have them, but some participants were unable to provide an example while others provided an example that was deemed invalid. It is reasonable to question whether participants who failed to give valid examples had a similar level of understanding of the phenomena in question to those who succeeded.

To probe this last matter, we conducted a one‐way ANOVA on the frequency ratings provided by only those 84 participants (13 five‐year‐olds, 22 seven‐year‐olds, 23 nine‐year‐olds, and 26 young adults) who provided an example of IAM with retrieval context, that is, who showed clearly that they had a grasp of the spontaneous nature of IAMs. We found that the frequency ratings by 5‐year‐olds (*M* = 3.77, *SD* = 1.01), 7‐year‐olds (*M* = 2.82, *SD* = 1.05), 9‐year‐olds (*M* = 3.17, *SD* = .78), and adults (*M* = 3.92, *SD* = .98), as well as the main effect of age group, *F*(3, 80) = 5.84, *p* = .001, *η* ^2^ = .19, and outcomes of Tukey post hoc tests, were virtually the same as those reported on the full sample in the results section. The only difference was that the ratings by 5‐year‐olds were now higher than those by 7‐year‐olds, *p* = .028. While this indicates that frequency ratings were not affected substantially by whether participants were able to report a valid example of an IAM or mind‐pop, future research should examine the issue more rigorously by conducting extended interviews to ensure that participants do indeed understand the phenomena they are being questioned about.

In conclusion, the findings of the present study suggest that it is feasible to interview children about their involuntary memories and elicit meaningful responses from them, which opens up interesting avenues for future research. As already noted, researchers could explore whether children's growing awareness of involuntary memories, particularly IAMs, contributes to the development of their declarative metamemory regarding the role of reminders in memory retrieval. Additionally, it should be possible to use the interview method to examine developmental changes in children's monitoring of other kinds of involuntary cognitions, including mind‐wandering, daydreaming, and spontaneous thoughts about the future (e.g., imaginings of planned events, hypothetical events, and hoped‐for events; Caza & Atance, 2019; McCormack et al., 2019). Like IAMs and mind‐pops, such experiences are reported by adults as happening frequently (see Cole & Kvavilashvili, 2019), which underscores the importance of studying their prevalence and development in children and adolescents. Examining age‐related changes in metamemory for a broad range of involuntary thoughts could help to shed light on the development, causes, and consequences of these ubiquitous but neglected everyday phenomena.

## Appendix

The memory interview questions (underlined text was for children whereas text in parentheses was for adults):

Q1. First of all, do you think you have a good memory?

***Probing for IAMs***

*Have you noticed that sometimes you remember something on purpose, for example, when you come home and your mother asks you what you did in school. You have to try to remember what happened at school and tell her about it. Or, perhaps your friend asks you what program you watched on TV last night and you try to remember the name of the TV program. (OK, the next thing I want to ask you about is two different types of memory. Have you noticed that sometimes you remember something on purpose, for example, when you meet a friend who asks you what you did last weekend. To answer this question you have to deliberately remember what happened last weekend.)*

*I am sure this has happened to you quite often, am I right? However, sometimes memories just pop into our head unexpectedly even when we are not trying to remember anything. For example, you may see a balloon in front of someone's house and this may remind you of how you played with a red balloon at your friend's (your nephew's) birthday party. Or, you may see an elephant on TV and this may remind you of your trip to the zoo (India) where you saw an elephant*.

Q2. Do you think this happens to you sometimes as well? Do memories ever just pop into your head even when you are not trying to remember anything?

Q3. (If yes to Q2) Please tell me about a time when a memory just popped into your head. What happened? What did you remember?

Q4. (If yes to Q2) How often do you think memories just pop into your head? Never? Very rarely? Occasionally? Quite often? Or every day?

***Probing for mind‐pops***

*But, sometimes something might pop into your head that is not a memory about anything that happened to you like your friend's (nephew's) birthday party. Instead, what pops into your head is just a single word, an image of something or a song or music. For example, today I was having a cup of tea and thinking about my friend when suddenly the word “lolly pop man” popped into my head and I was quite surprised because my friend is not a lolly pop man. Also, yesterday I was brushing my teeth and thinking about a film that I watched when suddenly I started singing “Happy Birthday to You” even though it was not anybody's birthday*.

Q4. Do you think this happens to you sometimes as well?

Q5. (If yes to Q4) OK, I am very interested to hear about what sorts of things pop into your head. First of all, have you ever had words or names pop into your head?

Q6. (If yes to Q5) Please tell me about a time when a word or name just popped into your head. What happened? What did you remember?

Q7. (If yes to Q5) How often do you think words and names just pop into your head? Never? Very rarely? Occasionally? Quite often? Or every day?

Q8. (If yes to Q4) Have you ever had an image or picture of something just pop into your head? *For example, an image of your school (university building), an image of Spiderman, or maybe an image of an ice‐cream just pops into your mind even though you were not trying to remember anything*.

Q9. (If yes to Q8) Please tell me about a time when an image or picture of something just popped into your head. What happened? What did you remember?

Q10. (If yes to Q8) How often do you think images or pictures just pop into your head? Never? Very rarely? Occasionally? Quite often? Or every day?

Q11. (If yes to Q4) Have you ever had songs or music just pop into your head?

Q12. (If yes to Q11) Please tell me about a time when a song or some music just popped into your head. What happened? What did you remember?

Q13. (If yes to Q11) How often do you think songs or music just pop into your head? Never? Very rarely? Occasionally? Quite often? Or every day?

***Soliciting explanations of mind‐pops***

Q14. Why do you think that things like words, pictures, and songs might just pop into your mind?
